# Optimizing Rare Disease Gait Classification through Data Balancing and Generative AI: Insights from Hereditary Cerebellar Ataxia

**DOI:** 10.3390/s24113613

**Published:** 2024-06-03

**Authors:** Dante Trabassi, Stefano Filippo Castiglia, Fabiano Bini, Franco Marinozzi, Arash Ajoudani, Marta Lorenzini, Giorgia Chini, Tiwana Varrecchia, Alberto Ranavolo, Roberto De Icco, Carlo Casali, Mariano Serrao

**Affiliations:** 1 Department of Medical and Surgical Sciences and Biotechnologies, “Sapienza” University of Rome, 04100 Latina, Italy; dante.trabassi@uniroma1.it (D.T.); carlo.casali@uniroma1.it (C.C.); mariano.serrao@uniroma1.it (M.S.); 2 Department of Brain and Behavioral Sciences, University of Pavia, 27100 Pavia, Italy; roberto.deicco@unipv.it; 3 Department of Mechanical and Aerospace Engineering, Sapienza University of Rome, 00184 Rome, Italy; fabiano.bini@uniroma1.it (F.B.); franco.marinozzi@uniroma1.it (F.M.); 4 Department of Advanced Robotics, Italian Institute of Technology, 16163 Genoa, Italy; arash.ajoudani@iit.it (A.A.); marta.lorenzini@iit.it (M.L.); 5 Department of Occupational and Environmental Medicine, Epidemiology and Hygiene, INAIL, Monte Porzio Catone, 00078 Rome, Italy; g.chini@inail.it (G.C.); t.varrecchia@inail.it (T.V.); a.ranavolo@inail.it (A.R.); 6 Headache Science & Neurorehabilitation Unit, IRCCS Mondino Foundation, 27100 Pavia, Italy; 7 Movement Analysis Laboratory, Policlinico Italia, 00162 Rome, Italy

**Keywords:** gait analysis, rare diseases, cerebellar ataxia, data balancing, data augmentation, generative artificial intelligence, inertial measurement unit, generative artificial network, conditional tabular generative artificial network, synthetic minority oversampling technique

## Abstract

The interpretability of gait analysis studies in people with rare diseases, such as those with primary hereditary cerebellar ataxia (pwCA), is frequently limited by the small sample sizes and unbalanced datasets. The purpose of this study was to assess the effectiveness of data balancing and generative artificial intelligence (AI) algorithms in generating synthetic data reflecting the actual gait abnormalities of pwCA. Gait data of 30 pwCA (age: 51.6 ± 12.2 years; 13 females, 17 males) and 100 healthy subjects (age: 57.1 ± 10.4; 60 females, 40 males) were collected at the lumbar level with an inertial measurement unit. Subsampling, oversampling, synthetic minority oversampling, generative adversarial networks, and conditional tabular generative adversarial networks (ctGAN) were applied to generate datasets to be input to a random forest classifier. Consistency and explainability metrics were also calculated to assess the coherence of the generated dataset with known gait abnormalities of pwCA. ctGAN significantly improved the classification performance compared with the original dataset and traditional data augmentation methods. ctGAN are effective methods for balancing tabular datasets from populations with rare diseases, owing to their ability to improve diagnostic models with consistent explainability.

## 1. Introduction

Clinical studies targeting populations with rare diseases often face significant methodological challenges, particularly regarding the high risk of beta error due to small sample sizes [1] that limits the validity of the results. This problem is particularly pronounced in gait analysis research [2], where laboratory settings and inherent technical complexities further complicate data collection and interpretation [1].

Hereditary cerebellar ataxia represents a group of rare, highly debilitating neurodegenerative disorders affecting a relatively small proportion of the population. It is characterized by impaired coordination, balance, and gait [3,4,5,6]. Understanding and accurately identifying movement abnormalities in people with hereditary cerebellar ataxia (pwCA) is crucial for clinical management, prediction and monitoring of disease progression, and potential therapeutic interventions. In terms of spatiotemporal and kinematic parameters, increased step width, reduced ankle joint range of motion, increased gait variability, impaired foot positioning, abnormal lower limb muscle activation, and abnormal pelvic rotation have been observed to distinguish pwCA from normal gait patterns and other neurological gait disturbances [7]. Ataxic gait is also characterized by a loss of coordination between the upper and lower regions of the body, resulting in higher upper body oscillations and a lack of local trunk stability, causing the latter to generate perturbations while walking [7,8,9,10,11,12].

Inertial measurement units (IMUs) proved to be non-invasive, accurate, and objective tools for assessing the motor abnormalities associated with cerebellar ataxia [13,14,15,16,17,18,19]. IMUs allow to detect gait features by measuring the acceleration of the trunk while walking [20]. Furthermore, the analysis of the trunk acceleration provides subordinate gait indexes that reflect individuals’ dynamic balance [21,22,23,24] and trunk instability during gait [22,23], thus enhancing the number of useful features to be included in gait datasets. Particularly, the short-term largest Lyapunov’s exponent (sLLE), the step length coefficient of variation (CV_steplength_), and the harmonic ratio (HR) have been described as trunk acceleration-derived gait indexes that characterize the trunk abnormalities of pwCA when compared to healthy subjects, reflecting local dynamic stability, variability, smoothness, and symmetry of trunk behavior, respectively.

In recent years, machine learning (ML) classifiers have emerged as valuable tools for distinguishing pathological individuals from healthy subjects based on gait data [25,26]. ML technologies are used to analyze complex datasets and reveal patterns that would be difficult to detect using traditional observational or statistical methods [27,28,29,30,31]. Automatic classification of gait abnormalities using ML algorithms, combined with gait analysis using IMUs, could allow for rapid and clinically meaningful assessment of gait abnormalities in people with movement disorders [26,32,33,34,35,36,37].

Although significant progresses have been made in this field, researchers may encounter methodological challenges, particularly in terms of data collection. The lack of large sample cohorts makes the training of effective predictive models difficult, as sample numbers are often unbalanced, affecting the accuracy and generalization of ML model results [38].

To address this challenge, the field of ML has developed advanced strategies [39,40]. Class balancing techniques, such as random sampling [41], synthetic minority oversampling technique (SMOTE) [42,43], and other methodologies [44], have shown promise in reducing data disparities through improved ML model training [45]. For instance, these approaches have already been used in a variety of research contexts, including medical diagnosis, gait and image analysis [46,47,48,49,50], and have shown significant improvements in disease detection and clinical outcome prediction [51,52]. 

Furthermore, the use of data augmentation and balancing techniques not only increases the quality of training datasets but could also reveal valuable insights hidden within the data. Therefore, our study aimed to investigate the effects of advanced data augmentation techniques, such as generative adversarial networks (GANs) [53], on gait datasets associated with hereditary cerebellar ataxia. The specific aim is to assess the effectiveness of these methods in terms of classification metrics performances compared with traditional sample balancing techniques, such as undersampling and oversampling.

The primary hypothesis of our work is that generative techniques, with their inherent ability to model and produce synthetic data, that do not significantly deviate from the original distributions, will outperform conventional methods in terms of effectiveness and accuracy, providing a superior diagnostic classification model for rare diseases.

## 2. Materials and Methods

**Figure 1:**
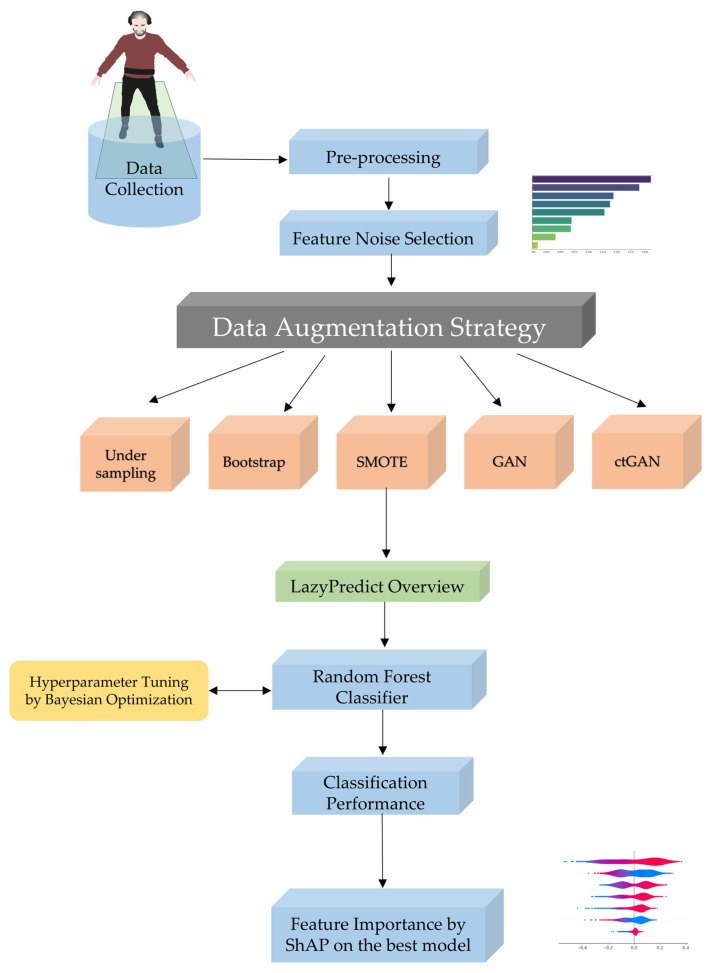
**Machine learning and data augmentation strategy.** A flowchart demonstrating the methodological strategy to enhance machine learning classification in the context of uncommon disease detection. It starts with data collection and progresses to preprocessing and feature noise selection to assure data quality. To overcome dataset imbalance, undersampling, bootstrapping, SMOTE, GAN, and ctGAN were used. LazyPredict package was used for an initial assessment of candidate models. The random forest classifier was then chosen for the classification task, with hyperparameter tuning performed via Bayesian optimization, and its performance was measured using known criteria. Finally, ShAP analysis increased the model’s explainability, ensuring transparency and understanding of how features influence predicted outcomes.

Figure 1 summarizes the workflow for the methods used in this study. The details are explained in the following subsections. 

### 2.1. Gait Data Acquisition

#### 2.1.1. Subjects

In total, 30 pwCA, aged 51.6 ± 12.2 years, 13 females and 17 males, and 100 age and gait speed matched healthy subjects (HS), aged 57.1 ± 10.4 years, 60 females, 40 males, were enrolled. All individuals were recruited at the Academic Neurorehabilitation Unit of the Traumatic Orthopedic Surgical Institute (ICOT) in Latina, Italy, from July 2021 to February 2023. The diagnosis and clinical features of all individuals are shown in Appendix A, Table A1. Disease severity was assessed using the Scale for the Assessment and Rating of Ataxia (SARA) [54,55]. Because pwCA may exhibit extracerebellar signs affecting gait performance, participants with gait impairment caused by extracerebellar symptoms (spasticity, polyneuropathy, cognitive impairment [MMSE score > 24], oculomotor abnormalities, and visual deficits according to the Snellen visual acuity test) were excluded. We only included people who could walk without assistance and had gait issues that were purely cerebellar at the time of their initial evaluation, based on a larger cohort of pwCA from a rare diseases center [56,57,58].

Subjects with gait-influencing diseases, such as peripheral neuropathies, clinically defined osteoarthritis, or joint replacement, were excluded from the HS group after an anamnestic and clinical examination of joint pain levels and range of motion.

In conformity with the Declaration of Helsinki, both pwCA and HS provided informed consent prior to the experimental procedure. The study was approved by the local ethics council (CE Lazio 2, protocol number 0139696/2021).

#### 2.1.2. Procedures

Gait data were collected using a single IMU (BTS GWALK, BTS, Milan, Italy) placed at the L5 level through an ergonomic velcro belt and connected to a laptop via Bluetooth. The sensor embeds a triaxial accelerometer (16 bit/axes), a triaxial magnetometer (13 bits), and a triaxial gyroscope (16 bit/axis). Linear trunk accelerations and angular velocities in the anterior–posterior (AP), mediolateral (ML), and vertical (V) directions were recorded at a sampling rate of 100 Hz [59] using the ‘Walk+’ protocol of the G-STUDIO software (BTS G-STUDIO, BTS, Milan, Italy). Both pwCA and HS were asked to walk along a 30 m long and about 3 m wide corridor at their own pace. Because we aimed at investigating natural locomotion, we provided only broad and qualitative instructions, allowing participants to determine their own gait speed without external sensory inputs. Specifically, we instructed subjects to begin walking at the end of the calibration procedure included in the ‘Walk+’ protocol, maintain a steady gait, and stop at the end of the pathway. The corridor floor was covered in linoleum, with no visible pavement joints or demarcation lines, and indirect lighting was distributed evenly along the pathway. Participants were instructed to familiarize themselves with the procedure by walking along the trail prior to the experiment. No adverse events were recorded during the procedures. 

To guarantee a steady-state walking assessment, we removed the first and last two strides from each 30 m walk. Gait trials with at least 20 consecutive accurately recorded strides [60,61,62], were included in the analysis. The following spatiotemporal and kinematic gait parameters were extracted using the G-STUDIO software: stance phase, single support phase, double support phase, swing phase, gait speed, cadence, stride length, pelvic tilt, pelvic obliquity, and pelvic rotation. 

As the trunk acceleration–derived gait indexes, the harmonic ratio (HR), % determinism and % recurrence within the recurrence quantification analysis (RQAdet and RQArec, respectively), the step length coefficient of variation (CV), and the short–term largest Lyapunov’s exponent (sLLE) were calculated in the three spatial directions using MATLAB software (MATLAB R2022a 7.4.0, MathWorks, Natick, MA, USA). For HR calculation in each acceleration direction, 20 harmonics were calculated for each subject based on stride time. Using a discrete Fourier transform, the trunk accelerations during each stride were separated into single sinusoidal waveforms. HR in the AP and V directions (HRAP and HRV, respectively) were calculated as the ratio of the sum of the first 10 even harmonics to the sum of the first 10 odd harmonics. HR in the ML direction (HRML) was calculated by adding the amplitudes of the odd harmonics and dividing by the sum of the amplitudes of the even harmonics. Noise signals were eliminated using a high-pass filter with a cutoff frequency of 20 Hz. HRs were calculated for each stride and averaged over a steady walk to obtain a mean HR [22].

HR was calculated as follows:

$${HR}_{AP,V}=\frac{{\sum_{i}A}_{i*2}}{\sum_{i}A_{i*2-1}}$$

$${HR}_{ML}=\frac{\sum_{i}A_{i*2-1}}{{\sum_{i}A}_{i*2}}$$

where *Ai* is the amplitude of the first 20 even harmonics and A_2i−1_ indicates the amplitude of the first 20 odd harmonics.

The step length CV was calculated as follows:

$${CV}_{step\;length}=100\;SD/mean$$

where mean is the mean step length and SD is the standard deviation across the entire step length for each subject. The step length is more variable as the CV*_step length_* increases [22].

RQA is a nonlinear data analysis approach that reveals patterns and the structure of dynamical systems. After reconstructing a recurrence plot, RQAdet and RQArec were calculated using the nearest-neighbor method by embedding acceleration and angular velocity data in a range of embedding dimension (m) 2–10; m = 5 was considered the optimal value based on false neighbors analysis conducted using Rtol = 17 and Atol = 2. Time delay (τ) was calculated based on the first minimum of the average mutual information function (AMI) using a range of τ 7–18. A time delay of 10 samples for the first minimum of the AMI function was used in this study. RQAdet reflects how often a trajectory repeatedly revisits similar state space locations (time dependent) and is quantified as the percentage of recurrent points in the diagonal line structures parallel to the main diagonal lines [22].

RQAdet was calculated as follows:

$$RQAdet=\frac{\sum_{l=lmin}^{N}lP(l)}{\sum_{l=1}^{N}lP(l)}$$

where P(l) is the frequency distribution of the lengths l of the diagonal lines in the recurrence matrix (how many patterns have length l).

RQArec reflects how often a trajectory visits similar locations in the state space and is calculated as the percentage of recurrent points in the recurrence matrix.

RQArec was calculated as follows:

$$RQArec=\frac{1}{N^{2}}\Sigma_{i,j=1}^{N}R_{i,j}$$

where N is the number of datapoints in the recurrence matrix; Ri,j are the elements of the recurrence matrix *R* where each element is either 0 or 1; i, j are indices ranging from 1 to *N*, representing the position of points in the time series.

The sLLE reflects gait local dynamic stability as the average logarithmic rate of divergence between the system’s trajectory and its nearest neighboring trajectory. When trajectories converge, the observed system tends to have local dynamic stability, while divergence indicates local dynamic instability. 

The accelerations were time-normalized to obtain 100 data points per stride. No filtering was applied to the accelerations to avoid the loss of spatiotemporal fluctuations and nonlinearities, thus excluding the effects of the time duration of the data series on LLE estimation.

The short-term largest finite-time Lyapunov exponent according to Rosenstein’s algorithm for short time series using the Lyaprosen toolbox (Matlab) for nonlinear time-series analysis was computed for each acceleration direction over the considered strides in each trial. The original data and delayed copies were juxtaposed to reconstruct a multidimensional state space from the recorded one-dimensional time-series data. The dimensions of the reconstructed space state were determined using the false nearest neighbor method, whereas the time delay was determined using the first minimum of the AMI function [22,63].

Table A1 in Appendix A shows the resulting spatiotemporal characteristics and trunk acceleration-derived gait indexes.

### 2.2. Pre-Processing

In the ML preprocessing phase, particularly when dealing with unbalanced datasets at the outset, several critical steps are undertaken to prepare the data for subsequent analysis [64]. We first loaded and inspected the dataset to explore its structure and content, to identify and include only the variables that covered both classes of output. 

Next, the dataset was deeply analyzed. The process involved reviewing the initial data, determining column data types and consistency of variables, and identifying any null or missing values that could invalidate the analysis.

Outliers were identified based on the interquartile range (IQR). Once identified, outliers were replaced with the feature’s median. We replaced five outliers from the HS group. IQR was chosen for its tolerance of extreme values and suitability for variables that do not follow a normal distribution.

To avoid skewing patterns toward the majority class and to improve the performance and stability of the machine learning models [64], we plotted the distribution of classes, and normalized the data distribution using a Power Transformation on numerical features.

Because algorithms may depend on the magnitude of the input data [65], after data cleaning and transformation, the features were standardized to balance their weights into the classifier. 

#### Feature Selection

First, we performed correlation analysis to identify and remove collinear features, which are variables that, due to their strong correlation with one another, have the potential to affect the model by providing redundant information. Then, we used an ensemble-based technique with a random forest (RF) classifier, which is well known for its feature selection capabilities [66] to select the most relevant features within the dataset [67]. Because the minority class was small, we chose to perform feature selection across the entire dataset in order to preserve as much information as possible. To reduce the risk of overfitting and improve model robustness, we used a cross-validation strategy during feature selection and training.

To serve as a baseline, we also generated a synthetic feature known as *noise* based on a random number generator, with no predictive value. Any less important characteristic than the noise feature was considered unlikely to be relevant to our model and excluded. After training the classifier, we computed the feature importance scores, which indicate how each attribute contributed to improving the model’s performance. To provide clarity and assist with understanding, we ranked these features according to their importance scores, resulting in a clear hierarchy of feature relevance. Afterwards, we used an independent-samples *t*-test to determine whether the variables obtained from the feature selection process differed significantly between the two classes.

The top-ranking features identified through this approach were then assessed for inclusion in the final model. 

### 2.3. Data Balancing Strategies

We compared the effectiveness of five data balancing strategies, which are explained in detail below. Regarding the augmentation strategies, we chose to increase the sample based on a priori sample size calculation or by randomly raising up to 1000 instances. A priori sample size calculation resulted in a needed sample of approximately 100 subjects in each class to reliably detect an effect size of |δ| ≥ 0.51 [22], assuming a two-sided criterion for detection using α = 0.05 and a conservative power of 0.95 in a 1:1 ratio.

#### 2.3.1. Undersampling and Oversampling

The undersampling strategy attempts to equalize the class distribution by reducing the size of the more prevalent class to match the sample size of the rarer class. While successful at balancing, it has the potential drawback of eliminating data that could include significant information. 

Undersampling was implemented using the ‘RandomUnderSampler’ library from Python’s ‘Imbalanced-Learn’ package. This class describes an undersampling method that attempts to balance the distribution of groups by lowering the size of the majority class. It randomly deletes cases from the majority class until a desirable level of balance with the minority class is achieved. Consequently, observations from the control group were deleted, while maintaining all information pwCA.

Instead, we oversampled to increase the number of observations in the minority class using the ‘RandomOverSampler’ class from the same Python ‘Imbalanced-Learn’ module. This class focuses on raising the number of instances in the minority class by resampling and replacing until a desired balance between the classes is achieved. 

#### 2.3.2. Synthetic Minority Oversampling Technique (SMOTE)

To address the imbalance between the two classes, we applied the synthetic minority oversampling technique (SMOTE). From ‘Imbalanced-Learn’ package we used the class ‘SMOTE’ that creates synthetic minority class samples to balance the sample distribution. SMOTE generates synthetic samples from existing minority class as follows.

**Figure 2:**
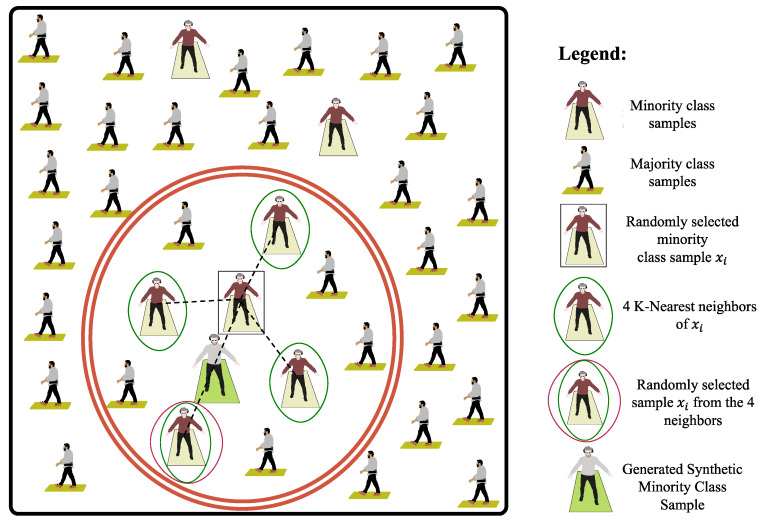
**SMOTE application in class balancing**. There are two distinct classes represented here: cerebellar ataxia subjects, as the minority class samples and healthy subjects, as the majority class samples. The majority class is represented by a greater number of subjects distributed across the field, whereas the minority class is represented by fewer individuals. SMOTE focuses on the minority class, which has fewer samples and is thus underrepresented in the dataset. A random sample is drawn from the minority class; this sample is designated as $x_{i}$. Its k-nearest neighbors are evaluated: the diagram depicts the four nearest neighbors of sample $x_{i}$ within the minority class, which are linked by dashed lines. These neighbors are in the feature space. One of the k-nearest neighbors is randomly chosen: a neighbor is chosen at random from among the four nearest neighbors. Interpolating between the original sample $x_{i}$ and the selected neighbor yields a new synthetic minority class sample.

For each minority class sample, the algorithm determines the k nearest neighbors within the same class. Then, one of these k neighbors is picked randomly, and a synthetic sample is created by performing a linear interpolation between that sample and the chosen neighbor [42]. This approach generates new instances that are similar but not identical to the original samples, hence increasing the variety of training data without merely replicating current instances. We initially set k = 4 for the identification of nearest neighbors, as shown in Figure 2, to keep an appropriate balance between the computational cost and the quality of the synthetic samples that have to be generated. 

#### 2.3.3. Generative Adversarial Network (GAN)

To address class imbalance, we also used a generative adversarial network (GAN). GAN represents a deep learning model that uses two neural networks, generator and discriminator, which work against each other to produce new synthetic instances of data [53]. Its purpose is to produce samples that can be added to the minority population. In our study, the generator was trained to create samples that closely resemble the distribution of actual data from HS and pwCA. A random ‘noise’ vector was used as a starter, and it runs through dense layers to represent the features of the data we want to recreate.

In contrast, the discriminator was trained to distinguish between real and synthetic data. Its function was to classify the input correctly and provide feedback to the generator on how effective the imitation was. During training, the generator and discriminator were trained in alternating directions; the generator increased its ability to imitate real data, whereas the discriminator developed its capacity to differentiate real data from fakes. This competitive training process was repeated until the generator produced data that the discriminator could not differentiate from the real data.

**Figure 3:**
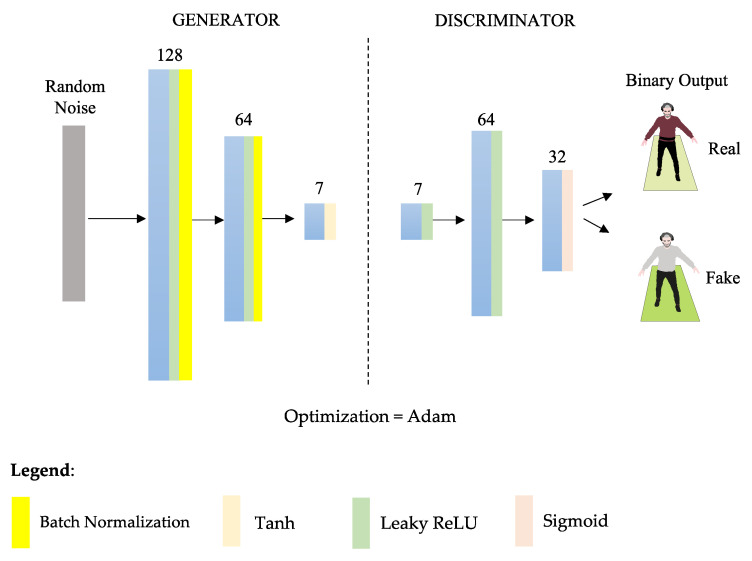
**GAN architecture.** Starting with the generator, the first layer after the random noise vector is a dense layer, which is fully connected and has 128 neurons. Following that, we have LeakyReLU as the activation function, which allows some negative values to ‘leak’ through, potentially avoiding the problem of dead neurons during training, with an alpha of 0.01 defining the slope of the negative part of the activation function. The subsequent batch normalization layer normalizes each batch’s input to keep the mean close to zero and the standard deviation close to one. This helps to stabilize the training and is widely used in GANs. The final layer of the generator contains as many neurons as there are features to be generated and employs the activation function tanh to produce the generator’s output, which are the synthetic features. In contrast, the discriminator’s first layer contains 64 neurons. The last layer contains a single neuron with a sigmoid activation function. This is because the discriminator is responsible for determining whether the data are real (value close to 1) or synthetic/false (value close to 0). The discriminator is built with binary cross entropy as the loss function, an Adam optimizer with a specified learning rate, and a beta parameter that handles the gradient’s exponential moving average decay.

To build and compile the neural model, we used TensorFlow and the Keras API in Python. The custom architecture that was used to generate new synthetic data for underrepresented pwCA is depicted in Figure 3. The newly generated observations were then added to the original dataset.

#### 2.3.4. Conditional Tabular Generative Adversarial Network (ctGAN)

Conditional tabular generative adversarial network (ctGAN) is a variation of GAN that allows for the generation of synthetic data conditioned on specific class labels or features and is specifically designed to handle tabular data [68].

**Figure 4:**
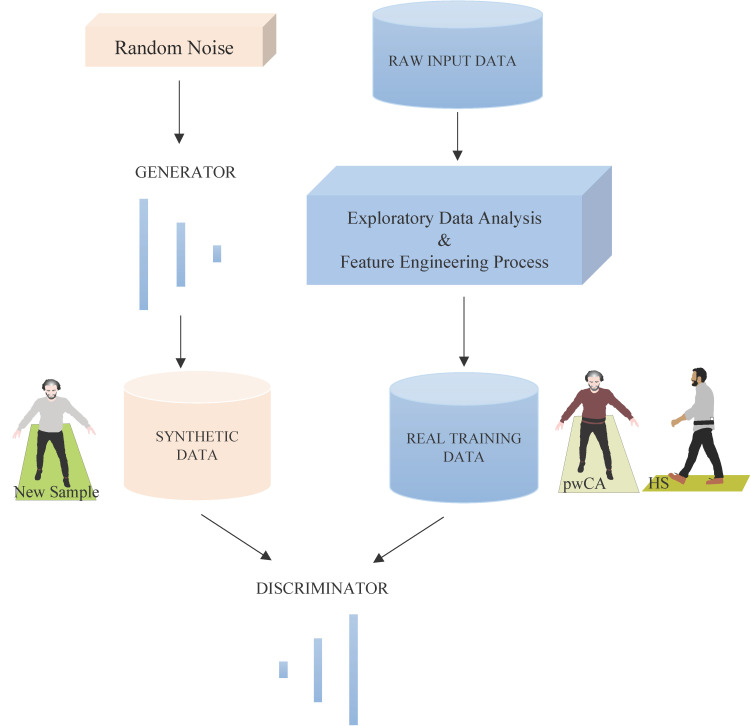
**Working process of a ctGAN**. The process begins with inputting random noise into the generator. This noise serves as a seed for creating new data samples. The generator takes this random noise and attempts to generate new synthetic data that closely resemble the distribution of the original training data. The generator gradually learns to produce more realistic data. The generator produces synthetic data, which should be indistinguishable from real data once the ctGAN is fully trained. The GAN discriminator is responsible for distinguishing between real training data and synthetic data produced by the generator. It provides feedback to the generator on the quality of the synthetic data. In addition to the synthetic data, the discriminator receives real training data. Exploratory data analysis and feature engineering processes are used to ensure that the training data is in the proper format and contains the necessary features to effectively train the discriminator. This represents the generator’s successfully generated synthetic sample, which the discriminator is unable to distinguish from real data The ctGAN is trained using an adversarial process in which both the generator and the discriminator iteratively improve themselves.

ctGAN, like a conventional GAN, uses dense layers to map a random noise vector onto synthetic data. The generator learns to generate data that closely resembles the distribution of real data, as illustrated in Figure 4.

We first introduced a conditional layer that contains conditional information. This enabled the network to generate data for our two distinct classes, pwCA and HS.

To stabilize the training and prevent the issue of dead neurons, we used batch normalization and LeakyReLU activation functions.

The discriminator, conversely, consisted of a series of dense layers that attempted to discriminate between real and synthetic data (Figure 4). LeakyReLU activation functions were used to enable tiny gradients when the unit is inactive to promote nonzero gradients during training. Batch normalization with a sigmoid activation function was implemented to improve the ability of the discriminator to distinguish real and synthetic data.

### 2.4. ML Classification Algorithm

The datasets were analyzed using Python’s LazyPredict package to determine which supervised classification algorithm performed the best in terms of accuracy and computational cost. As a result, RF provided the optimum trade-off in terms of computational cost versus forecast accuracy. This technique was then developed, using k-fold cross validation with k = 4, considering the hyperparameters to be handled to maximize performance. We employed a stratification strategy while splitting the data for cross-validation to guarantee that both classes were consistently represented in each fold. Stratification was used to preserve class balance throughout all folds, hence increasing the model’s robustness and generalizability.

In addition, we chose Bayesian optimization, which is a hyperparameter selection strategy that employs a probabilistic model, such as a Gaussian process, to model the performance function and then use this model to predict performance with various hyperparameter combinations [69]. Bayesian optimization selects a set of hyperparameters from an initial distribution. It tests the model’s performance in that combination and uses the results to update the initial distribution, transforming it into a post hoc probability distribution. It then chooses the next set of hyperparameters based on the revised distribution, usually the one that maximizes predicted acquisition.

In this study, the hyperparameters analyzed were as follows:

- The total amount of trees in the forest; an interval between 50 and 500 was chosen as a compromise between searching for improving model performance and computational costs, implying that the optimal number of trees between 50 and 500 was sought.
- The tree’s maximum depth; a range between 2 and 20 was specified to analyze trees of varying depths, from very simple (2 levels) to highly complicated (20 levels). Deeper trees than 20 levels could have captured more complicated associations, but they would also increase the risk of overfitting in training data.
- The smallest number of samples necessary to split an internal node; values ranging from 2 to 10 were chosen, thus limiting the minimum number of samples required in a node to be considered for subsequent splits, hence preventing overfitting.
- The minimal number of samples needed to form a leaf node; we specified the range of minimum samples required in a leaf node in a range from 1 to 10 in order to optimize the bias/variance trade-off.

#### 2.4.1. Performance Metrics

- Accuracy was defined as the proportion of accurately positive and negative predicted cases based on the total number of cases. It was computed as

$$Accuracy=\frac{TP+TN}{TP+FP+FN+TN}$$

where TP = true positives, TN = true negatives, FP = false positives, and FN = false negatives.

Recall was defined as the proportion of positive cases accurately detected by the model. For a specific class, it was calculated as

$$Recall=\frac{TP}{TP+FN}$$

- Precision is the proportion of correctly predicted positive cases to the total predicted positives. For each class, it was calculated as

$$Precision=\frac{TP}{TP+FP}$$

- F1 Score represents the harmonic mean of precision and recall, yielding a single score that balances both criteria. It is particularly beneficial when you need to balance precision and recall. It was calculated as

$$F1\;Score=2\;*\;\frac{Precision\;*\;Recall}{Precision\;+\;Recall}$$

- Log loss is a performance metric that measures the penalty based on the likelihood that the model assigns to the actual correct class.

It is considered as a ‘soft’ metric because it penalizes the confidence of incorrect predictions. A smaller log loss suggests a model that performs better, with log loss = 0 reflecting ideal log loss.

$$Log\;Loss=-\frac{1}{N}\sum_{i=1}^{N}\left[y_{i}\;*\;log{(p}_{I}\right)+\left(1-y_{i}\right)\;*\;log(1-p_{i})]$$

where

*N* is the number of observations, $y_{i}$ is a binary indicator (0 or 1) that indicates whether the class label for the $i_{th}$ observation is correct, and $p_{i}$ is the model’s predicted probability that the $i_{th}$ observation is in the positive category.

- Receiver Operating Characteristic Curves (ROCs) were plotted and their Area Under the Curve (AUC) was calculated. AUC is an overall performance metric of the classifier, with values ranging from 0 to 1, with 1 representing a flawless model that accurately separates all positive cases from negative ones [70].

#### 2.4.2. Consistency and Explainability Analysis

To assess whether the feature distributions in original unbalanced and the data generated by ctGAN were consistent, the Kolmogorov–Smirnov (KS) test was employed by analyzing the cumulative distribution functions (CDFs) of two samples to detect significant differences. The KS statistic is the highest absolute value of the difference between the CDFs of the two samples. The test was performed at a 95% confidence interval.

Furthermore, to improve model explainability and better understand the weight of individual features in the classification model with the greatest performance measure, we used a Shapley value analysis. We applied SHAP (sHapley Additive exPlanations) to interpret machine learning model predictions. SHAP calculates each feature’s impact on predictions while accounting for feature interactions [71]. The Shapley value of a feature represents its average marginal contribution over all possible feature combinations. As a result, SHAP values indicate not only whether a feature is important or not, but also how it influences the model output.

A bee swarm plot, displayed as a violin plot of SHAP values, was constructed specifically to increase the metric explainability of the best model, by depicting the distribution of each feature’s impact on model output, with each point representing an observation from the dataset. 

## 3. Results

### 3.1. Feature Selection Results

**Figure 5:**
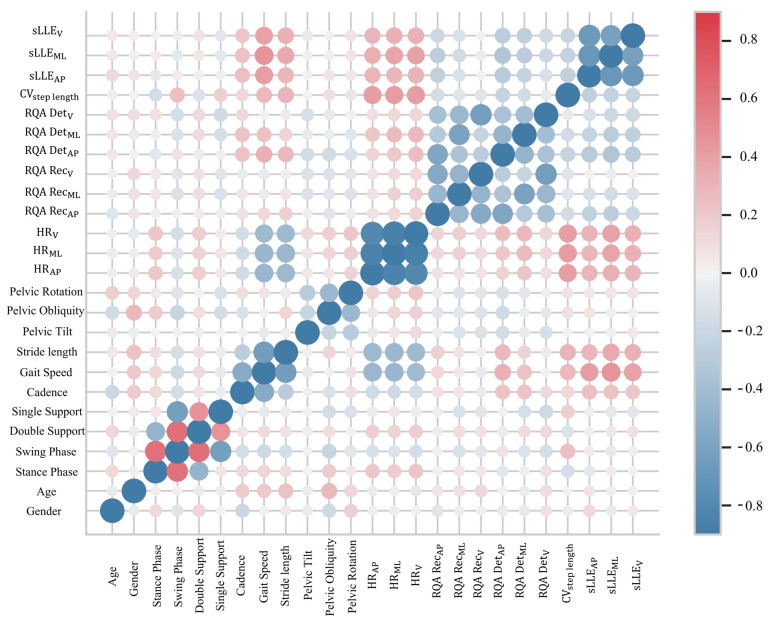
**Correlation heatmap.** The heatmap obtained using the Seaborn library’s relplot function displays the correlations between the initial features. Each cell in the matrix represents the partial correlation between two variables, as shown by the variable names on the x- and y-axes. The color of the cell indicates the direction and strength of the correlation: red for positive and blue for negative. The size of the circle within the cell represents the magnitude of the correlation coefficient. A threshold of 0.5 was chosen to determine which characteristics should be included in the dataset. HR, harmonic ratio; sLLE, short-time largest Lyapunov’s exponent; RQArec, %recurrence in recurrence quantification analysis; RQAdet, %determinism in recurrence quantification analysis; CV*_steplength_*, coefficient of variation of step length; AP, ML, V, anterior–posterior, mediolateral, and vertical direction of the acceleration signal, respectively.

We found a significant relationship between stance phase and other variables like swing phase, double support, and single support (Figure 5). Given the complementary nature of stance phase to swing phase and its ability to capture relevant information about support phases, we chose to include only stance phase in our final dataset. We decided to include only ${HR}_{AP\;}\;$ and ${sLLE}_{AP}\;$ in the final dataset due to their clinical relevance [22,23,72]. As a result of the feature selection procedure, we selected eight variables from an initial analysis of 25 gait parameters (see Appendix A, Table A1). 

**Figure 6:**
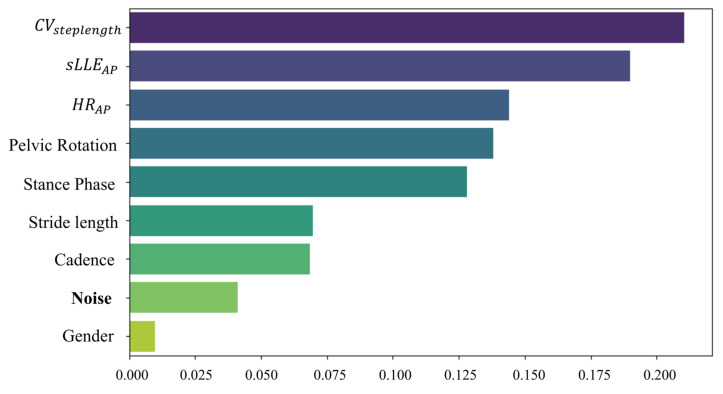
**Feature Importance plot.** Each bar represents a feature used in the RF model, and the length of the bar indicates how important that feature is when making predictions. Importance is typically calculated based on how much each feature reduces the impurity of the division. The Noise feature, which was introduced to help determine which features to keep, acts as a baseline. If the real features are similar or less important than the noise, they may not contribute significantly to the model’s predictions and can be removed in the next iteration.

Figure 6 shows the results of our selection algorithm, which uses RF methodology. 

The analysis using RF revealed that the classifier performed well for seven of the eight previously identified features. The categorical variable ‘gender’ was excluded because it had no greater importance than a dummy ‘noise’ feature, which was included in the model for comparison purposes. The addition of ‘noise’ was intended to further refine the selection, retaining only features with a high information value for our classification model. As a result, we decided to use a smaller set of seven features in the subsequent supervised machine learning algorithm to assess its effectiveness in classifying gait abnormalities in pwCA.

The selected features significantly differentiated between pwCA and HS, as shown in Table 1. The Supplementary Materials include a detailed description of the selected gait indexes (Supplementary Material, Table S1). 

#### Supervised ML Classification Metrics

Table 2 shows the classification metrics used to evaluate the effectiveness of each sample balancing technique. 

The classification achieved the highest accuracy (0.90) with the ctGAN dataset (*n* = 200), indicating that synthetic data generation via ctGAN produced a training sample that allowed the RF model to generalize more effectively during testing. As shown in Table 2, our RF model outperformed the initial unbalanced datasets, as well as datasets modified with conventional techniques such as under- and over-sampling and the SMOTE method, when trained with balanced data, particularly when obtained through ctGAN.

The results for each class in the models are described in Table 3. 

The initial unbalanced dataset had a good precision for the pwCA class (0.80) but a relatively low recall (0.35), indicating that, despite good recognition of true positives, many pathological cases were not identified. For HS, both metrics were remarkably high (precision = 0.93, recall = 0.93), indicating the unbalanced model correctly recognized the true negatives.

The use of data balancing strategies significantly improved recall for the pathological class, with ctGAN performing the best, particularly at N = 200.

### 3.2. Consistency and Explainability Results

The results of the Kolmogorov–Smirnov (KS) test are described in Table 4. No significant differences in the distributions of the variables in the original and ctGAN-generated dataset were found. 

**Figure 7:**
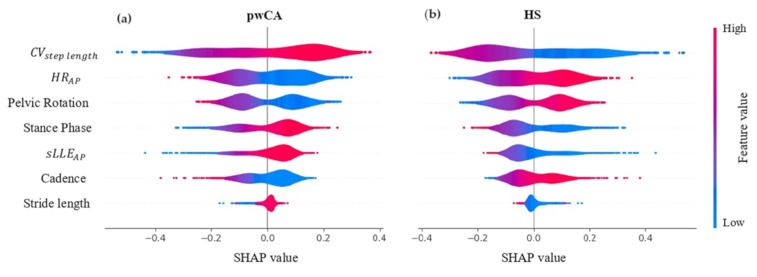
**SHAP value plots.** The x-axis shows the SHAP value associated with each feature. A SHAP value indicates the impact of a feature on model output. Positive values increase the prediction, towards a more positive outcome, whereas negative values decrease the prediction towards a more negative outcome. The color denotes the feature value, with red indicating high values and blue indicating low values. For example, if a feature (in red) has high values and is associated with positive SHAP values, the predicted outcome tends to improve as its value increases. The two graphs (**a**,**b**) show how the importance and effects of the features differ between the two models, pwCA and HS. Some features have a stronger positive or negative impact in one model than in the other.

ShAP findings are shown in Figure 7. ${CV}_{step\;length}$, HR, and pelvic rotation were the most important variables in the classification model.

## 4. Discussion

We proposed to investigate the effectiveness of data augmentation techniques to improve the gait classification performance of machine learning procedures in a cohort of subjects with a rare disease, such as pwCA.

The main findings of the study can be summarized as follows:

1. Testing various dataset balancing strategies revealed that the analyzed generative artificial intelligence methods outperformed traditional techniques in terms of the classifier’s performances.
2. ctGAN was the best method for balancing sample classes when classifying a rare condition such as cerebellar ataxia based on inertial sensor gait tabular data (Table 4).
3. The synthetic data generated by the ctGAN model appeared to be reliable because of their strong similarity with the original data.
4. The synthetic data generated by the ctGAN model yielded sound and explainable results regarding the impact of gait variables on the classification model.

In our study, data balancing methods were applied on a solid preprocessed dataset of gait features robustly selected through a filter, embedded, and domain-expertise-based feature selection procedure [25], to optimize our model by using just the most significant predictors, hence improving the ability of the classifier to discover characteristics patterns related to the pathology of interest [73].

By carefully selecting features, we not only streamline the model to focus on essential data, but we also limit the risk of overfitting and increase computing efficiency. The RF algorithm was used as the classification model to train and test the initial and generated datasets obtained through sample class balancing strategies. We chose to use RF algorithm by using the LazyPredict package which engenders several ML algorithms and identifies the best performing one according to the specific dataset. Notably, RF has been already used in other studies investigating classification of subject based on pathology, resulting in excellent classification performances [25,74,75,76,77,78]. 

Before proceeding to manage the minority class, to avoid potential biases, due to the structure of our unbalanced data, we subsampled the majority class to test the performances of the classifier after the sampling procedure which, as expected, showed poor performances (Table 3).

Afterwards, we adopted different balancing strategies to increase the sample size of the minority class to see how the classifier performed on the different datasets. Notably, we used the random oversampling and SMOTE strategies [42] which both proved to be reliable methods for gait data balancing [79,80]. Both strategies resulted in increased recall in pwCA (Table 4) and F1 Score in both pwCA and HS groups (Table 3). However, whereas both strategies were able to improve the model’s ability to recognize the minority class, they were not able to improve the performances of the majority class (Table 4). 

In an attempt to further increase the performances in both classes, we trained on datasets obtained through GAN methods. GAN is a deep learning model that uses two neural networks, generator and discriminator, which work against each other to produce new synthetic instances of data [53]. ctGAN, is a recently proposed variation of GAN that allows the generation of synthetic data conditioned on certain class labels or features [68]. Although GAN has been widely used for visual data [81], very recently GAN methods have proved to also provide reliable results for tabular datasets, including gait analysis [82,83,84,85,86]. Notably, it has demonstrated that GAN algorithms were able to classify the ON–OFF fluctuations of gait in Parkinson’s disease by using a single inertial sensor [82]. 

We found the ctGAN outperformed the unconditioned GAN and SMOTE techniques in classification tasks for all the metric performances (Table 3). A possible explanation of the higher performance of ctGAN relies on its inherent structure that embeds a priori binary factor that conditions the generation of the synthetic data. Generative neural networks can generate synthetic data with real-world statistical properties, allowing datasets to be balanced without introducing significant bias [87]. They also contribute to better minority class representation, lowering the risk of overfitting and increasing model generalization. These advantages have been demonstrated in various studies, including that of Lee et al. They also confirmed that in most cases, generative methods outperform traditional methods [87]. For this reason, SMOTE has almost always proven to be less effective than more sophisticated generation methods. SMOTE may perform worse than GAN in a scenario with 30 to 100 observations due to its linear interpolation method, which may not capture the complexity and variability of the original data in higher-dimensional environments [88]. GANs, which generate synthetic data that mimics real data distributions, can provide greater diversity and representation, which is crucial for enriching a limited dataset without significant distortions. Therefore, the superior performance of ctGANs can be attributed to their ability to generate more realistic synthetic data, as they incorporate conditional constraints that better capture the underlying data distribution. This ability allows ctGANs to produce high-fidelity samples that enhance model training, leading to improved generalization and classification accuracy.

We found that the best performances for ctGAN as well as all for all the other augmentation balancing strategies were obtained from a dataset with a sample size of 200 observations. In this study, we performed an a priori sample size analysis based on the discriminating ability of the variables that were included in the model to define the proper sample to obtain through augmentation techniques. This finding suggests that, when generating synthetic data, the nature of the variables in the specific population, as well as the characteristics of the original dataset, should be accomplished. 

Our findings align with previous studies showing the effectiveness of generative models in medical data augmentation [89,90]. Particularly, the use of ctGANs has shown to be feasible in several applications, such as electroencephalography [91,92]. 

However, this is the first time that ctGAN has been used as a strategy to increase the dimensions of the minoritarian class on a tabulated dataset obtained from a single inertial sensor in the analysis of pathological patients. The best results from the dataset obtained through ctGAN reflect the ability of this neural network to generate synthetic data conditions on specific etiquette. ctGAN, in fact, is designed to generate synthetic data based on specific dimensions or characteristics, and its ability to focus on specific conditions or minority classes aids in the production of more representative and balanced data, improving the model’s addition quality [68]. Furthermore, due to its conditional structure, it has the potential to be more effective at learning and replicating internal complexities from a data set than a traditional GAN. This leads to the generation of synthetic data that better retains the statistical structures and relationships present in the original dataset. 

Given the black box nature of the ctGAN algorithm, we sought to improve the model’s reliability and explainability of the ctGAN results [93]. Firstly, we conducted a Kolmogorov–Smirnov analysis to compare the gait features between the real and the synthetic datasets (200 N). We found no statistically significant differences between the sample distributions for all the gait features (Table 4, indicating that the ctGAN augmentation algorithm does not alter the statistical distribution of the investigated gait features. Secondly, we used a Shapley values-based strategy [94] to test the impact of each gait feature on the classification model in order to understand the most important gait variables in class identification and to ensure that the results were consistent with the known gait abnormalities of pwCA [7]. As shown in Figure 7, the Shapley values, related to each gait feature for the two classes (i.e., pwCA and HS), showed that ${CV}_{step\;length}$, HR, and pelvic rotation are the most impacting features on the classification model, in line with existing evidence. Regarding the findings on trunk acceleration-derived gait indexes, ${CV}_{step\;length}$ and HR represent parameters reflecting the variability of gait behavior and the smoothness and symmetry of trunk acceleration patterns, respectively, and have been reported to correlate with history of falls in pwCA [22,72,95,96,97]. The violin shape of sLLE, a measure of local dynamic gait instability [23,24], revealed that higher sLLE values had a lower impact on pwCA classification than HR and CV. This is consistent with recent findings indicating that sLLE is more useful as a responsive outcome measure of local dynamic stability to assess the effectiveness of interventions than for classification purposes in pwCA, due to its inherent dependence on gait speed [23,24]. Regarding the kinematic and spatiotemporal gait features, we found that pelvic rotation values have a positive impact on the classification of both classes. This supports the hypothesis of reduced pelvic mobility in response to chaotic upper trunk behavior and impaired trunk-lower limb coordination during gait [7,12,22,98]. 

Our findings are consistent with previous research using similar machine learning techniques to classify pathological gaits. Phan et al. (2020) used random forest (RF) to quantify gait ataxia in patients with neurological disorders using wearable sensors, with promising classification accuracy [32]. Mirelman et al. (2019) used machine learning techniques to detect subtle mobility changes in various stages of Parkinson’s disease, proving the effectiveness of RF models combined with inertial sensor gait data [26]. These studies support our decision to use RF for classification and suggest that data balancing techniques improve the classification performance of machine learning models.

Furthermore, Yang et al. (2022) observed that using the synthetic minority oversampling technique (SMOTE) enhances models’ ability to detect gait abnormalities in subjects with neurological disorders [99]. Our findings using ctGAN support these claims, but they also imply that ctGAN may have additional advantages over other methods such as SMOTE, particularly when dealing with small and imbalanced datasets.

Although the performance metrics reflected excellent classification results, a potential limitation of the study is the absence of clinical variables in the dataset, which could have improved the classification metrics or the generation of synthetic data. 

Furthermore, we employed the entire dataset for feature selection, as justified by the relatively small minority class size (pwCA). Although this decision may raise the risk of overfitting, we mitigated it by using cross-validation techniques during feature selection and model training. This strategy enabled us to use all relevant information while maintaining the generalizability of the model. 

However, the model’s generalizability suffers due to the small dimensions of the minority class. Although we employed data balancing approaches, such as ctGAN, to increase the amount of samples in the minority class, it is important to note that constructing synthetic data points from the variance obtained in the limited real-world dataset may have limitations. Although data balancing procedures might help enhance model performance, they can additionally emphasize specific aspects of the original dataset, increasing the possibility of overrepresenting some specific characteristics of the specific sample. Furthermore, the synthetic data generated may not fully align with the complexity and diversity of real data, limiting the ability of the model to generalize to previously unseen data. These issues should be considered while interpreting our study’s findings.

Future studies with greater sample sizes should investigate this strategy to ensure the proposed models’ robustness and dependability.

## 5. Conclusions

The proposed method for balancing the dataset to avoid a beta type error in classification using generative artificial intelligence was suitable to a typical unbalanced dataset of features from a population with a rare disease. Furthermore, using Kolmogorov–Smirnov’s analysis and methods like SHAP may improve the clarity and interpretability of metrics generated by machine learning applications, allowing clinicians to better understand the classification process and translate the findings into clinical decisions.

Particularly, ctGAN outperformed the other data balancing techniques, with significant improvements in accuracy, precision, recall, and F1 Score, as well as a decrease in log loss and an increase in ROC AUC. The generated dataset reflected the characteristics of the original samples, as well as the impact of the variables in the classification model reflected the evidence in the trunk acceleration-based gait analysis literature in pwCA, with pelvic rotation, HR_AP_, and CV_step length_ showing the highest importance in discriminating between pwCA and HS.

The findings of this study may provide insights into the distinctive gait patterns of pwCA, but also provide practical strategies for improving the robustness and accuracy of ML classifiers in rare disease contexts. Through this research, we aim to pave the way for more effective diagnostic tools and interventions for pwCA. Future research should investigate the use of ctGANs in other rare disorders, as well as long-term clinical outcomes, to validate these preliminary findings.

## Figures and Tables

**Table 1:** Independent sample *t*-test.

	pwCA	HS	*p*	Cohen’s d
	Mean (SD)	Mean (SD)		
**Stance Phase**	64.66 (3.31)	61.62 (4.94)	<0.001	0.640
**Cadence**	97.92 (17.93)	99.39 (13.23)	0.003	0.412
**Stride length**	1.17 (0.19)	1.24 (0.18)	0.003	0.496
**Pelvic rotation**	5.19 (2.46)	3.97 (2.62)	<0.001	0.578
${HR}_{AP}$	1.84 (0.57)	2.45 (0.68)	<0.001	1.033
${CV}_{step\;length}$	43.23 (16.14)	23.63 (12.52)	<0.001	1.311
${sLLE}_{AP}$	0.58 (0.22)	0.40 (0.21)	<0.001	1.253

HR, harmonic ratio; CV, coefficient of variation; sLLE, short–time largest Lyapunov’s exponent; AP, anterior–posterior direction of the acceleration signal.

**Table 2:** Overall classification performance.

	Accuracy	Recall	F1 Score	Log loss	ROC AUC
	Mean (SD)				
**Initial Unbalanced**	0.79 (0.2)	0.79(0.1)	0.75 (0.3)	0.42 (0.3)	0.87 (0.2)
**Undersampling**	0.77 (0.4)	0.77 (0.3)	0.78 (0.2)	0.49 (0.3)	0.89 (0.1)
**Oversampling**	0.83 (0.3)	0.82 (0.4)	0.83 (0.4)	0.38 (0.2)	0.89 (0.2)
**SMOTE (N = 200)**	0.80 (0.1)	0.80 (0.2)	0.79 (0.1)	0.40 (0.1)	0.87 (0.2)
**SMOTE (N = 1000)**	0.75 (0.2)	0.74 (0.1)	0.75 (0.2)	0.41 (0.3)	0.86(0.1)
**GAN (N = 200)**	0.83 (0.1)	0.83 (0.2)	0.79 (0.1)	0.42 (0.2)	0.83 (0.4)
**GAN (N = 1000)**	0.82 (0.2)	0.83 (0.1)	0.81 (0.3)	0.44 (0.1)	0.86 (0.2)
**ctGAN (N = 200)**	0.90 (0.1)	0.88 (0.2)	0.88 (0.1)	0.35 (0.1)	0.90 (0.1)
**ctGAN (N = 1000)**	0.81 (0.3)	0.80 (0.1)	0.79 (0.1)	0.40 (0.2)	0.85 (0.2)

SMOTE, synthetic minority oversampling technique; GAN, generative adversarial network; ctGAN, conditional tabular generative adversarial network; N, sample size after balancing strategy implementation.

**Table 3:** Comparison of classification metrics in pwCA and HS classes.

	pwCA		HS	
	Precision	Recall	Precision	Recall
	Mean (SD)		Mean (SD)	
**Initial Unbalanced**	0.78 (0.2)	0.37 (0.1)	0.91 (0.3)	0.91 (0.2)
**Undersampling**	0.55 (0.4)	0.88 (0.2)	0.95 (0.1)	0.72 (0.1)
**Oversampling**	0.83 (0.4)	0.60 (0.3)	0.84 (0.3)	0.92 (0.2)
**SMOTE (N = 200)**	0.72 (0.3)	0.55 (0.2)	0.81 (0.2)	0.93 (0.1)
**SMOTE (N = 1000)**	0.58 (0.2)	0.60 (0.3)	0.82 (0.1)	0.83 (0.3)
**GAN (N = 200)**	0.90 (0.2)	0.40 (0.1)	0.98 (0.2)	0.80 (0.2)
**GAN (N = 1000)**	0.84 (0.1)	0.5 (0.2)	0.82 (0.3)	0.96 (0.1)
**ctGAN (N = 200)**	0.85 (0.1)	0.75 (0.1)	0.92 (0.2)	0.92 (0.2)
**ctGAN (N = 1000)**	0.83 (0.2)	0.6 (0.2)	0.82 (0.2)	0.88 (0.1)

**Table 4:** Kolmogorov–Smirnov test results.

Gait Parameter	KS	*p*-Value
**Cadence**	0.22	0.09
**Stride length**	0.18	0.23
${sLLE}_{AP}$	0.17	0.35
${HR}_{AP}$	0.15	0.48
**Pelvic rotation**	0.09	0.92
**Stance phase**	0.08	0.98
${CV}_{step\;length}$	0.06	0.99

## Data Availability

The data presented in this study are available on request from the corresponding author and stored in a password-protected PC located in the Department of Medico-Surgical Sciences and Biotechnologies, University of Rome Sapienza.

## Supplementary Materials

The following supporting information can be downloaded at: https://www.mdpi.com/article/10.3390/s24113613/s1, Table S1: Pseudocodes.

## Appendix A

**Table A1:** Sample characteristics, gait stability indexes, and spatiotemporal values.

	CA Subtype	#
**Subjects** (n)	ACD	5
	SAOA	4
	MSA-C	2
	SYNE1	1
	SCA-NDD	2
	SCA1	6
	SCA2	4
	SCA3	2
	SCA6	1
	SCA8	2
	SCA27b	1
	**pwCA (n = 30)**	**HS (n = 100)**
**Age** (years)	51.60 (12.73)	57.08 (10.40)
**SARA** (n)	12.66 (4.68)	
**SARAgait** (n)	3.03 (1.19)	
**Falls** (n)	3.43 (4.48)	
**Gait speed** (m/s)	0.97 (0.25)	1.02 (0.24)
**Stance phase** (% Gait cycle)	64.66 (3.31)	61.62 (4.94)
**Swing phase** (% Gait cycle)	35.34 (3.31)	38.03 (3.36)
**Double support phase** (% Gait cycle)	14.70 (3.53)	12.29 (4.84)
**Single support phase** (% Gait cycle)	35.26 (3.83)	37.58 (5.42)
**Cadence** (steps/min)	97.92 (17.93)	99.39 (13.23)
**Stride length** (m)	1.17 (0.19)	1.24 (0.18)
**Pelvic tilt** (°)	3.05 (0.98)	2.99 (1.12)
**Pelvic obliquity** (°)	3.97 (3.99)	3.76 (4.47)
**Pelvic rotation** (°)	3.97 (2.62)	5.19 (2.46)
**HRap**	1.84 (0.57)	2.45 (0.68)
**HRml**	1.79 (0.45)	2.23 (0.54)
**HRv**	1.81 (0.45)	2.33 (0.62)
**RQA RECap** (%)	7.09 (10.32)	5.15 (8.57)
**RQA RECml** (%)	5.02 (5.58)	4.82 (6.68)
**RQA RECv** (%)	6.05 (10.17)	4.70 (6.34)
**RQA DETap** (%)	33.70 (26.65)	29.54 (26.35)
**RQA DETml** (%)	37.17 (24.21)	32.79 (25.73)
**RQA DETv** (%)	27.00 (26.14)	24.73 (21.50)
${CV}_{step\;length}$ (%)	43.23 (16.14)	23.63 (12.52)
**sLLEap** (1/s)	0.58 (0.22)	0.40 (0.21)
**sLLEml** (1/s)	0.36 (0.20)	0.25 (0.17)
**sLLEv** (1/s)	0.39 (0.20)	0.37 (0.25)

HR, harmonic ratio; RQA REC, recurrence of recurrence quantification analysis; RQA DET, determinism of recurrence quantification analysis; CV, coefficient of variation; sLLE, short–time largest Lyapunov’s exponent; AP, anterior–posterior direction of the acceleration signal; ML, mediolateral direction of the acceleration signal; V, vertical direction of the acceleration signal.
