# Supplementary material for: Optimizing Rare Disease Gait Classification through Data Balancing and Generative AI: Insights from Hereditary Cerebellar Ataxia
Source: Sensors (Basel). 2024 Jun 3;24(11):3613. doi: 10.3390/s24113613 (PMC11175240; doi:10.3390/s24113613)
Supplement: Supplementary file 1 [file sensors-24-03613-s001.zip › sensors-3027528-supplementary.pdf]

SUPPLEMENTARY MATERIAL

Pseudocodes.

Noise Feature Selection

1 Import the pandas, numpy, and RandomForestClassifier modules

2 Load a dataset from an Excel file

3 Set a random seed for reproducibility

4 Add a column 'Noise' to the data frame with random numbers

5 Separate the data frame into features (X) and output (y), excluding the 'Pathology' column from features

6 Initialize a RandomForestClassifier with 100 trees and a random state of 42

7 Fit the Random Forest model on the features and output

8 Get the importance of each feature

9 Create a Data Frame to display the feature importance’s, with columns 'Feature' and 'Importance'

10 Sort the Data Frame by 'Importance' in descending order

11 Print the sorted feature importance’s

12 Plot the feature importance’s using seaborn's barplot, with a figure size of (10, 6), a 'viridis' palette, and labeled axes and title

Smote

1 Install the imbalanced-learn package

2 Import SMOTE for oversampling, pandas for data manipulation, and Counter for counting object occurrences

3 Load a real training dataset from an Excel file

4 Separate the loaded dataset into features (X) and output (y), excluding the 'Pathology' column from the features

5 Count and print the occurrences of each class in the output dataset

6 Define a desired sample count per class and the number of neighbors for SMOTE

7 Initialize SMOTE with a sampling strategy for specified classes to reach the desired count (200 or 1000), set number of neighbors (4) , and a random state for reproducibility(42)

8 Apply SMOTE to resample the dataset, balancing specified classes

9 Create a new Data Frame from the resampled data, including the resampled features and output

10 Save the resampled training dataset to an Excel file

GAN

1 Import necessary libraries and modules

2 Load dataset from an Excel file

3 Separate the dataset based on 'Pathology' value

4 Define function to build generator model with parameters for hyperparameters
- Define model architecture with Sequential
- Add layers: Dense, LeakyReLU, Batch Normalization, and output layer based on hyperparameters

5 Define function to build discriminator model with parameters for hyperparameters
- Define model architecture with Sequential
- Add layers: Dense, LeakyReLU, and output layer based on hyperparameters
- Compile model with loss and optimizer using hyperparameters

6 Define function to build GAN combining generator and discriminator
- Set discriminator's trainable to False
- Combine generator and discriminator in Sequential model
- Compile GAN with loss and optimizer using hyperparameters

7 Define hyperparameters grids for tuning
- Learning rates for Adam optimizer
- Beta_1 value for Adam optimizer
- Number of neurons in layers for both generator and discriminator

8 Initialize variables to store best model and performance metric

9 For each combination of hyperparameters in the grid
- Set hyperparameters for generator, discriminator, and GAN based on current grid values
- Rebuild generator, discriminator, and GAN with current set of hyperparameters
- Train GAN with these hyperparameters
    - Alternate between training discriminator on real and generated data
    - Train generator via GAN, which has discriminator feedback loop disabled
- Evaluate GAN performance based on chosen criteria (e.g., discriminator accuracy, quality of generated samples)
- If current performance is better than previous best, update best model and performance metric

10 Use the best hyperparameter set for further processing
- Generate separate noise for 'HS' and 'CA' classes
- Generate synthetic data for 'HS' and 'CA' using the best generator model
- Create and concatenate Data Frames for synthetic data

ctGAN

1 Import necessary libraries and modules for CTGAN and data handling

2 Load dataset from excel files

3 Define the hyperparameters grid for tuning
CTGAN hyperparameters:
- Number of epochs
- Generator learning rate
- Discriminator learning rate
- Batch size
- Number of generator layers and neurons per layer
- Number of discriminator layers and neurons per layer
- Embedding size for categorical columns

4 Initialize variables to store the best model and performance metric

5 For each combination of hyperparameters in the grid
- Initialize CTGAN model with current set of hyperparameters
- Train CTGAN model on the dataset
- Evaluate CTGAN model performance based on chosen criteria
  # Criteria examples: model loss, quality of generated data, specific metrics for tabular data fidelity
- If current performance is better than previous best, update best model and performance metric

6 Use the best hyperparameter set for further processing
- Generate synthetic data using the best CTGAN model
- Optionally, validate the synthetic data quality or utility based on specific use cases or metrics
- Use or save the synthetic data as needed

<u>Random Forest Classifier</u>

1 Import necessary libraries including scikit-optimize for Bayesian optimization, pandas for data handling, and metrics from sklearn

2 Load training and testing datasets from Excel files

3 Separate features and output labels for both training and testing datasets

4 Define RandomForestClassifier model with a fixed random state

5 Define hyperparameter search space for the RandomForestClassifier
- Number of trees in the forest (n_estimators)
- Maximum depth of the trees (max_depth)
- Minimum number of samples required to split an internal node (min_samples_split)
- Minimum number of samples required to be at a leaf node (min_samples_leaf)

6 Initialize Bayesian optimization over hyperparameters with BayesSearchCV
- Use RandomForestClassifier as the estimator
- Specify hyperparameters search space
- Use 4-fold cross-validation
- Perform the search in parallel jobs
- Use accuracy as the scoring metric
- Set a random state for reproducibility

7 Fit the optimizer to the training data to find the best hyperparameter values

8 Predict output labels for the testing dataset using the optimized model

9 Calculate performance metrics including accuracy, recall, precision, F1 score, log loss
- Calculate precision for each class
- Use classification report to get detailed metrics including recall (sensitivity) per class
- Calculate macro and micro average metrics from classification report

10 Display the calculated performance metrics

11 Calculate ROC AUC score for binary classification tasks

12 Calculate and display confusion matrices for each class
- For each class, display the confusion matrix using seaborn's heatmap for better visualization.
